# Agreement between in vivo and ex vivo photon-counting CT measurements of subchondral trabecular bone features in patients with knee osteoarthritis

**DOI:** 10.1007/s00330-025-11948-9

**Published:** 2025-08-27

**Authors:** Camilla Toft Nielsen, Mikael Boesen, Marius Henriksen, Janus Uhd Nybing, Sophia Wiinberg Bardenfleth, Christian Kento Rasmussen, Mathias Willadsen Brejnebøl, Asbjørn Seenithamby Poulsen, Saber Muthanna Aljuboori, Kristine Ifigenia Bunyoz, Søren Overgaard, Anders Troelsen, Henning Bliddal, Henrik Gudbergsen, Felix Müller

**Affiliations:** 1 Department of Radiology, Copenhagen University Hospital - Bispebjerg and Frederiksberg, Bispebjerg Hospital, Copenhagen, Denmark; 2 The Parker Institute, Copenhagen University Hospital - Bispebjerg and Frederiksberg, Frederiksberg Hospital, Copenhagen, Denmark; 3 Department of Radiology, Copenhagen University Hospital - Herlev and Gentofte, Herlev Hospital, Herlev, Denmark; 4 Faculty of Health and Medical Sciences, Department of Clinical Medicine, University of Copenhagen, Copenhagen, Denmark; 5 Visual Computing, DTU Compute, Technical University of Denmark, Copenhagen, Denmark; 6 Department of Orthopaedic Surgery and Traumatology, Copenhagen University Hospital - Bispebjerg and Frederiksberg, Bispebjerg Hospital, Copenhagen, Denmark; 7 Department of Orthopaedic Surgery, Copenhagen University Hospital - Amager and Hvidovre, Hvidovre, Denmark; 8 Department of Public Health, Centre for General Practice, University of Copenhagen, Copenhagen, Denmark

**Keywords:** Photon-counting CT, Knee osteoarthritis, Bone morphology, Agreement

## Abstract

**Objectives:**

The aim of this study was to compare in vivo and ex vivo Photon Counting CT (PCCT) of subchondral bone features in patients with knee osteoarthritis (KOA).

**Materials and methods:**

Pre-surgery in vivo and post-surgery ex vivo PCCT of the tibial plateau from participants with severe KOA referred to arthroplasty surgery from January 2022 through September 2023 were compared. Linear regression and Bland-Altman plots were used to assess correlation and agreement between in vivo and ex vivo measures of bone volume fraction (BV/TV), trabecular thickness (Tb.Th.) and attenuation in healthy and sclerotic trabecular bone. Delineated areas of bone sclerosis were compared using the Dice coefficient and Hausdorff distance.

**Results:**

18 in vivo/ex vivo PCCT scans were included. Strong correlations were found for BV/TV, *R*^2^ = 0.82 and attenuation; healthy, *R*^2^ = 0.89, and sclerotic, *R*^2^ = 0.79, bone, while a moderate correlation was found for Tb.Th., *R*^2^ = 0.55. Bias for BV/TV and Tb.Th. was −4.1% and −0.598 mm, respectively, and −41.4 HU and −81.1 HU for healthy and sclerotic bone, respectively. A proportional bias was observed for Tb.Th. and BV/TV. There was excellent agreement between the segmentations of sclerotic areas (Dice coefficient = 0.91, Hausdorff distance = 0.11 mm).

**Conclusion:**

In patients with severe KOA, BV/TV and attenuation can be obtained with a high correlation and small bias between in vivo and ex vivo scans, while Tb.Th. showed moderate correlation and larger bias. Longitudinal studies using in vivo PCCT are feasible, but caution may be advised when measuring Tb.Th. The key OA feature of subchondral bone sclerosis is well translated from ex vivo to in vivo PCCT.

**Key Points:**

***Question*** *Bone changes occur with osteoarthritis development; the role of these changes is unclear, and no method for visualising bone microstructure in vivo exists.*

***Findings*** *Photon-counting CT showed a strong correlation between in vivo and ex vivo subchondral density measures, while a moderate correlation was found for trabecular thickness.*

***Clinical relevance*** *Photon-counting CT is feasible for in vivo longitudinal evaluation of bone in patients with knee osteoarthritis, allowing studies into the earlier stages of the disease.*

**Graphical Abstract:**

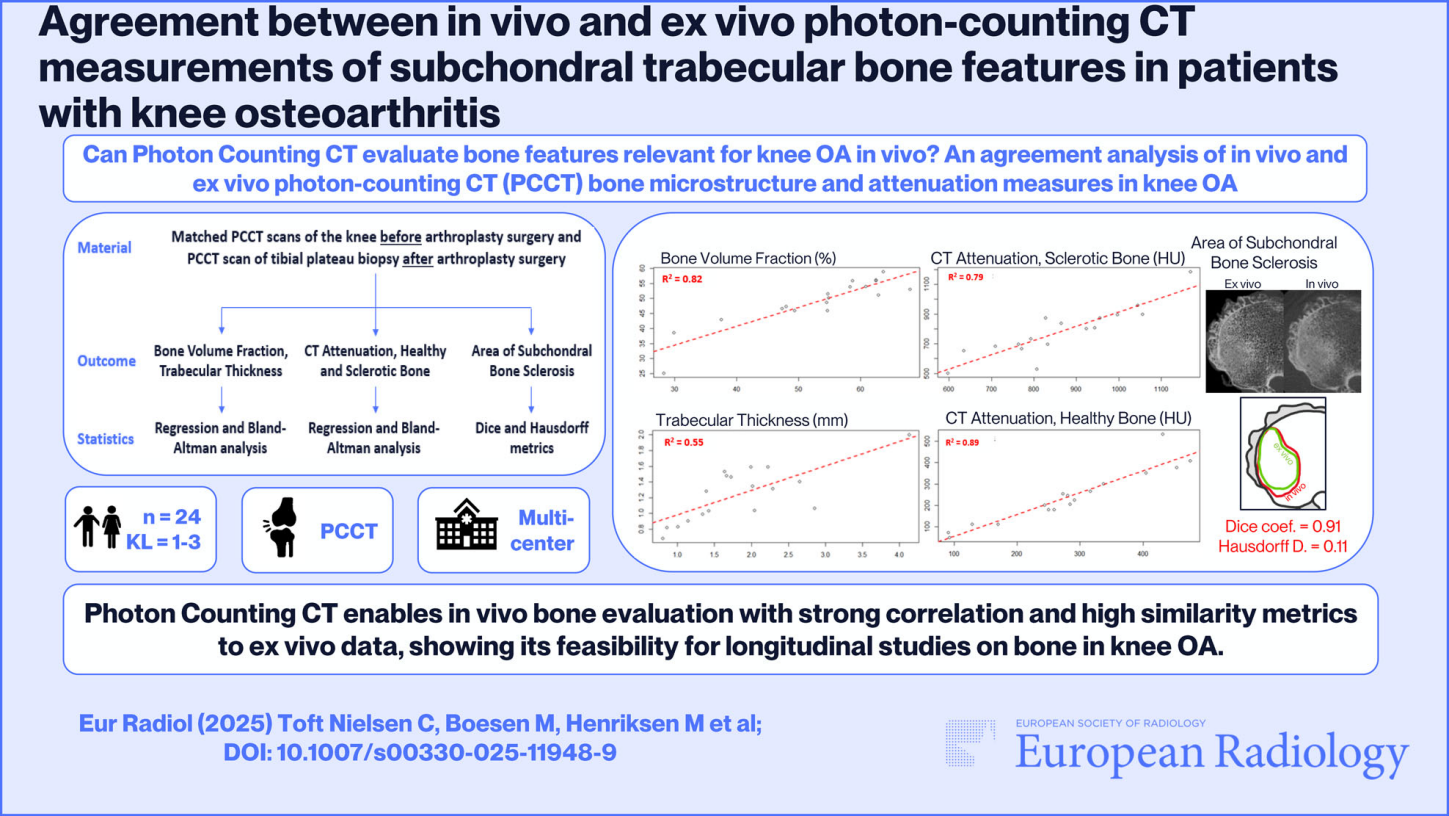

## Introduction

Bone morphology and density changes occur with disease initiation and progression in osteoarthritis (OA) [1–4]. On imaging such as radiographs and computed tomography (CT), these changes include subchondral bone sclerosis, bone cysts, and osteophyte formation. Many questions are still unanswered regarding the changes in bone microstructure seen in OA. One major contributor to this is the inability to study the structure of bone in vivo. This precludes longitudinal studies on the evolution of the bone microstructure with disease progression. There is a need for longitudinal studies to understand the pathological mechanisms that cause the structural bone changes that contribute to joint deterioration. Medications targeting bone formation, such as bisphosphonates and calcitonin, along with other bone-acting agents, such as strontium ranelate, have been investigated for their effects on pain relief and structural progression in OA to date without significant effect [5]. However, without an understanding of the role bone plays in OA and sensitive methods of tracking bone changes, it might be premature. If an effective disease-modifying OA drug targeting bone modulation were to become available, a non-invasive imaging modality would be invaluable for the longitudinal studies investigating its effects.

To date, conventional CT using clinically acceptable radiation doses does not have the resolution capabilities to accurately depict the trabecular structures of subchondral bone [6]. Therefore, studies investigating the structural changes in bone-related diseases rely mainly on surgical or cadaver specimens for ex vivo imaging using High-Resolution-peripheral Quantitative CT (HR-pQCT) or micro CT (μCT) [7]. Photon-Counting CT (PCCT) is superior to conventional CT in terms of effective spatial resolution [8]. It has nearly the same level of detail as HR-pQCT but with a lower level of contrast [6]. PCCT can produce Ultra-High-Resolution (UHR) imaging in vivo with fast scan time and no knee circumference constraints, ideal for studying bone in patients with knee OA. Even though ex vivo PCCT has produced imaging on par with μCT and HR-pQCT [9–11], it has not been shown that PCCT can depict bone microstructures in vivo in a clinic-like setting in patients with knee OA.

The question remains whether we lose important insights concerning bone features relevant to OA when we move from ex vivo to in vivo PCCT imaging. Therefore, the aim of the study was to investigate the correlation and agreement between in vivo PCCT and ex vivo PCCT subchondral bone volume fraction, trabecular thickness, trabecular attenuation, and area of subchondral sclerosis in patients with knee OA.

## Materials and methods

We included imaging data from a randomised trial (ClinicalTrials.gov Identifier: NCT05172843, The INKA trial) comparing weight loss and knee arthroplasty surgery on pain outcome in patients with severe knee OA and obesity. Ethical approval and informed consent were obtained (Health Research Ethics identifier: H-21035007), and the study was conducted according to the Declaration of Helsinki.

In this retrospective analysis of a prospective cohort, participants with knee OA and obesity who were referred to knee arthroplasty surgery in two centres in Copenhagen, Denmark, from January 2022 through September 2023 were invited to participate.

### Photon-counting CT

Participants included in the main trial had PCCT (Siemens Naeotom Alpha, Siemens Healthineers AG, Forchheim, Germany) of both knees at enrolment. Participants allocated to the surgery arm of the trial had tibial plateau biopsies collected during surgery, and these were immediately frozen and stored at −80 °C. Post-surgery, PCCT of the tibial plateau specimens was performed using the same PCCT scanner as the in vivo scans but at a greatly increased dose. The biopsies were scanned in frozen state and did not undergo any additional processing prior to imaging. The scans were performed using the UHR mode. Acquisition and reconstruction details included using Quantum Iterative Reconstruction, a tube potential of 120 kV, a matrix size of 1024 × 1024, a slice thickness of 0.2 mm, a FOV of 150 × 150 mm and quantum iterative reconstruction strength of 3. The volume computed tomography dose index (CTDIvol) was 12.83 mGy for the in vivo scan and 16.04 mGy for the ex vivo scans. An image reconstruction kernel, Br89, was used.

### Morphological bone parameters

Bone volume fraction (BV/TV) and trabecular thickness (Tb.Th.) were measured. First, the ex vivo scan was registered to the in vivo scan using ITK-SNAP (version 4.2.2, www.itksnap.org).

This was done by roughly aligning the scans manually and then performing ITK-SNAP’s automatic registration using a rigid transformation and mutual information as the image similarity metric. Linear interpolation was used to reslice the ex vivo scans to produce a volume with voxel-to-voxel correspondence with the in vivo scan. Second, a bounding box was manually placed in the trabecular bone, not involving the cortical bone plate of the registered scans. Third, thresholding, using the Otsu method, was applied to all voxels within the bounding box. Otsu is an adaptive thresholding method that has been shown to be superior compared to global thresholding when assessing the microstructure of bone [12]. Lastly, using a custom Python script and the Python package localthickness, BV/TV and Tb.Th. were calculated.

### Bone attenuation

The ex vivo scan was registered to the in vivo scan in the open-source software 3D Slicer (version 5.8.0, https://www.slicer.org/), using a set of manually placed corresponding anatomical Landmarks and affine rigid registration. To measure the attenuation in Hounsfield Units (HU), four circular regions of interest (ROI) measuring 6 mm in diameter were placed on an axial slice, two in the in vivo scan and two mirroring the placement in the registered ex vivo scan, The ROIs were manually placed in areas of healthy and sclerotic trabecular bone; ROI 1–4: (1) in vivo scan healthy bone, (2) in vivo scan sclerotic bone, (3) ex vivo scan healthy bone and (4) ex vivo scan sclerotic bone. The average HU in each ROI were calculated using the segment statistics application in 3D Slicer.

### Subchondral bone sclerosis area

Using the same registrations as for bone attenuation, manual segmentation of the area with sclerosis on the ex vivo and in vivo scans was done by one resident with collectively 3 years of clinical experience in rheumatology and radiology (C.N.), blinded to demographic and clinical data but not in vivo/ex vivo scan type. The segmentations were done on one axial slice, using the segment editor application in 3D Slicer, ensuring the chosen axial slice was under the cortical plate by cross-referencing the sagittal and coronal reformats.

### Statistical analysis

For the bone attenuation measures and morphological bone parameters, Linear regression and Bland–Altman plots on in vivo and ex vivo measurements were plotted to obtain correlation coefficients and measurements of bias, along with 95% limits of agreement using the statistical software R (version 2024.04.2+764, https://www.r-project.org/). Dice coefficients and Hausdorff distance compared the segmented areas of trabecular sclerosis to assess the similarity between measurements on in vivo and ex vivo scans using the segment comparator application available in the SlicerRT extension in 3D Slicer.

## Results

**Fig. 1:**
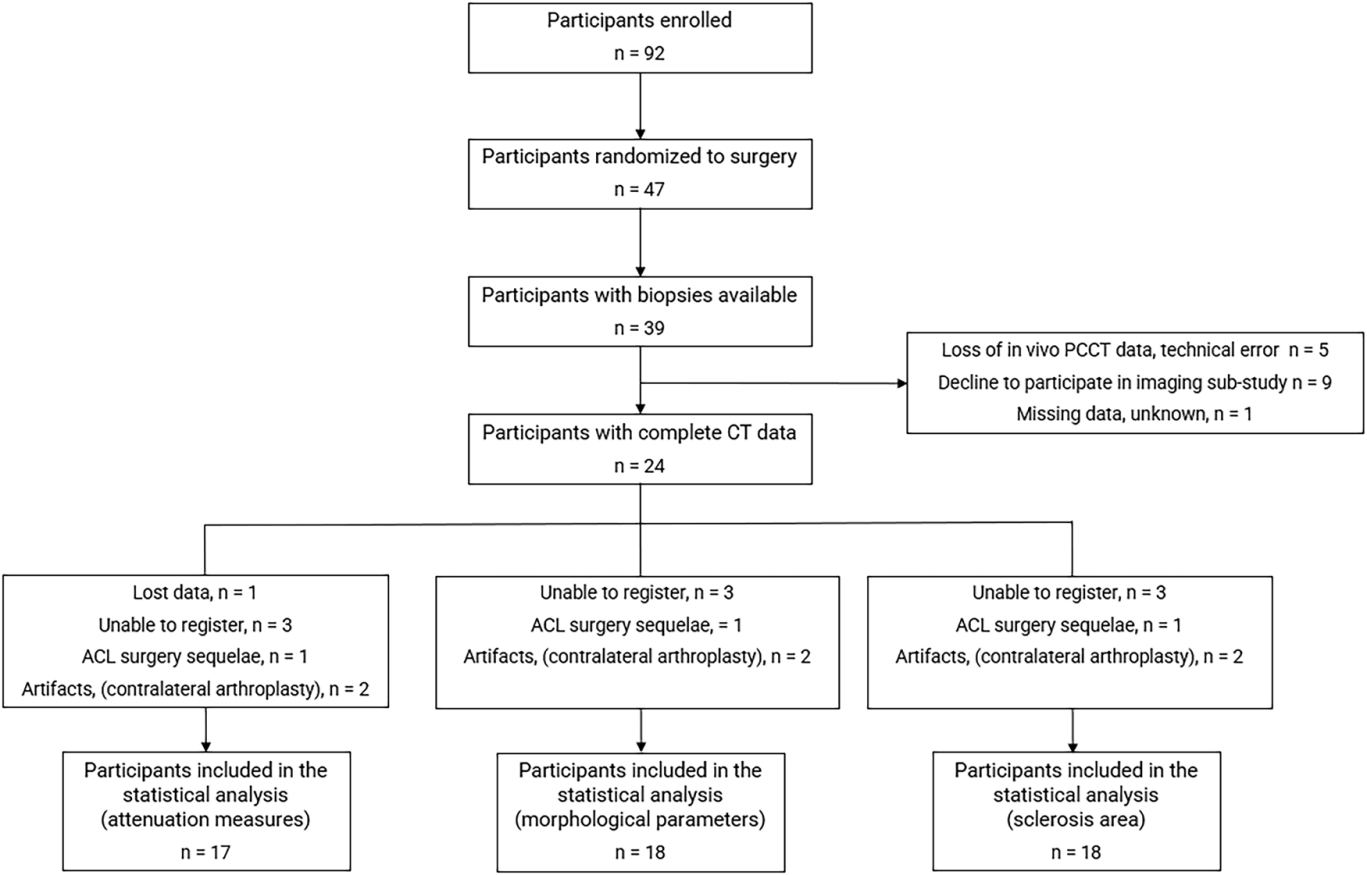
Flow diagram of study inclusion. Lost data: Registration data was lost for one in vivo/ex vivo data set prior to the attenuation analysis. ACL surgery sequelae: Participant excluded due to a screw in the tibia after anterior cruciate ligament (ACL) graft surgery

Of the 92 participants included in the main trial, 47 were allocated to the surgery arm. Both in vivo and ex vivo PCCT were available for 24 participants; participants’ demographics are presented in Table 1. Data from 17 participants were available and included in the attenuation analysis, 18 in the morphological analysis and 18 in the area subchondral sclerosis analysis. The flow diagram, including reasons for missing data, is presented in Fig. 1.

**Table 1:** Baseline participant demographics

	Total, *n* = 24
Female, *n* (%)	13 (54)
Age, years (± SD)	67 (± 7.86)
BMI, kg/m^2^ (± SD)	37 (± 4.52)
Kellgren-Lawrence Grade (KLG)	
KLG 2, *n* (%)	1 (4)
KLG 3, *n* (%)	6 (25)
KLG 4, *n* (%)	17 (71)

Values are means and ± standard deviations (SD) unless otherwise specified

### Morphological bone parameters

**Fig. 2:**
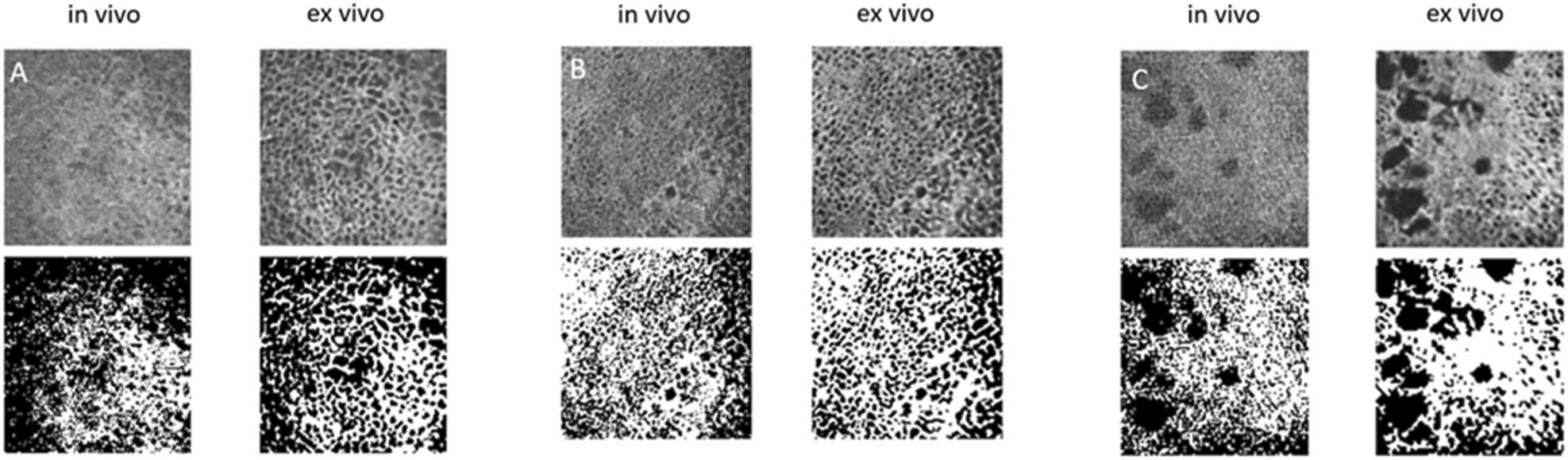
**A**–**C** Case examples of the top slice of the bounding box (top row) and corresponding slice with thresholding (bottom row). Left: in vivo, right: ex vivo

Three image examples of the bounding boxes and thresholding used to calculate the morphological bone parameters can be found in Fig. 2.

**Fig. 3:**
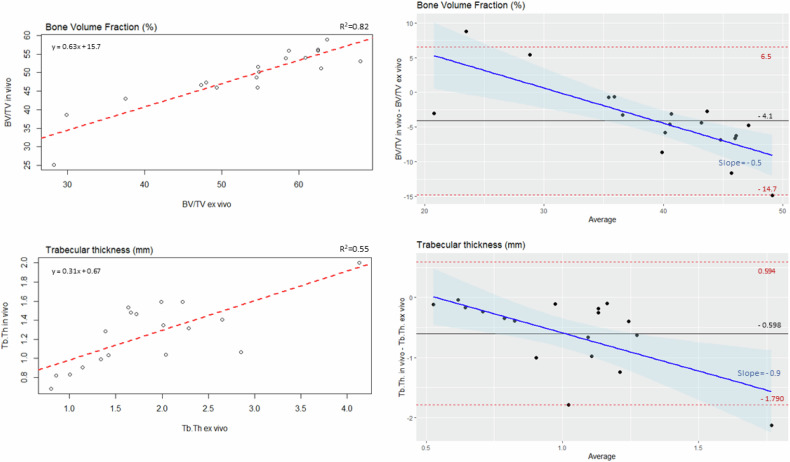
Linear regression (left) and Bland–Altman (right) plots for bone volume fraction (BV/TV) (top panel) and trabecular thickness (Tb.Th.) (bottom panel). Bland–Altman plots with mean difference (black line), 95% limit of agreement (dashed red lines) and regression fit (blue line) with 95% confidence interval (shaded blue area)

Linear regression and Bland–Altman plots for the in vivo and ex vivo BV/TV and Tb.Th. showed that the correlation between in vivo and ex vivo measures was high for BV/TV, *R*^2^ = 0.82 (b = 0.63) and moderate for Tb.Th., *R*^2^ = 0.55 (b = 0.31), Fig. 3. In the Bland–Altman plots, BV/TV showed a bias of −4.1% with 95% limits of agreement from −14.7% to 6.5% and for Tb.Th., a bias of −0.598 mm with 95% limits of agreement from −1.790 mm to 0.594 mm, i.e., lower values of the in vivo measures compared to ex vivo measure. For both BV/TV and Tb.Th., a proportional bias was observed, BV/TV with a slope of −0.5 (95% CI: −0.74; −0.27) and Tb.Th. with a slope of −0.9 (95% CI: −1.25; −0.58), indicating that the spread of the differences was systematically lower as a function of increasing average values, Fig. 3.

### Bone attenuation

**Fig. 4:**
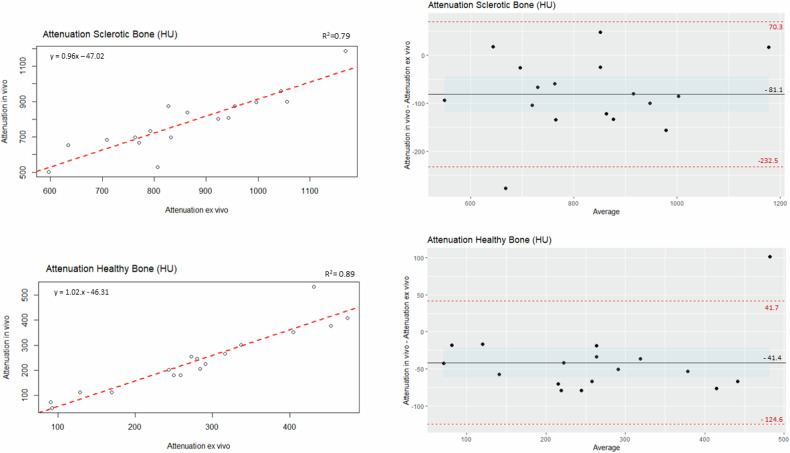
Linear regression (left) and Bland–Altman (right) plots for attenuation of sclerotic bone (top panel) and healthy bone (bottom panel). Bland–Altman plots with mean difference (black line) with 95% confidence interval (shaded blue area) and 95% limit of agreement (dashed red lines)

Linear regression and Bland–Altman plots for the in vivo and ex vivo attenuation measures for healthy and sclerotic bone showed that the correlation was high for both healthy and sclerotic bone, *R*^2^ = 0.89 (b = 0.1.02) and *R*^2^ = 0.79 (b = 0.96), respectively, Fig. 4. No trends were observed in the Bland–Altman plots, and a bias of −41.4 with 95% limits of agreement from −124.6 to 41.7 and −81.1 HU with 95% limits of agreement from −232.5 to 70.3 was observed for healthy and sclerotic bone, respectively. Again, the in vivo measures were, on average, smaller than the ex vivo measures.

### Subchondral bone sclerosis area

**Fig. 5:**
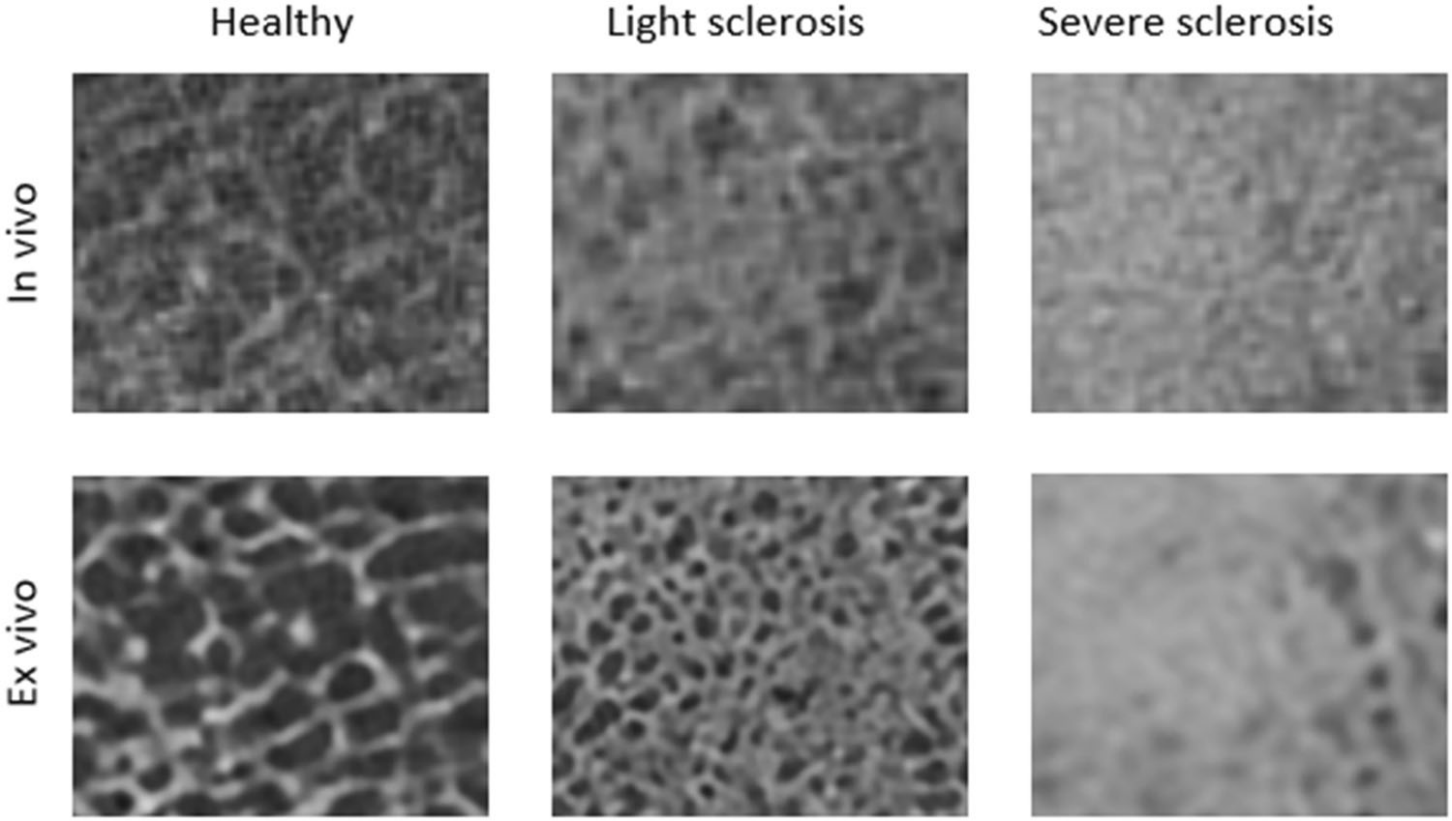
Case example from in vivo and ex vivo PCCT showing the different degrees of sclerosis observed. The in vivo and ex vivo examples are not from the same slice location

Descriptive: Studying the areas of subchondral sclerosis on the PCCT images, some distinct bone sclerosis patterns became evident. The bone could generally be divided into three patterns. First, the healthy bone with a clear trabecular pattern and dark marrow space. Second, areas where the trabecular structure was still visible, but the marrow space had a higher density. Third, a complete loss of trabecular structure to a high-density compact bone. Examples are shown in Fig. 5.

**Fig. 6:**
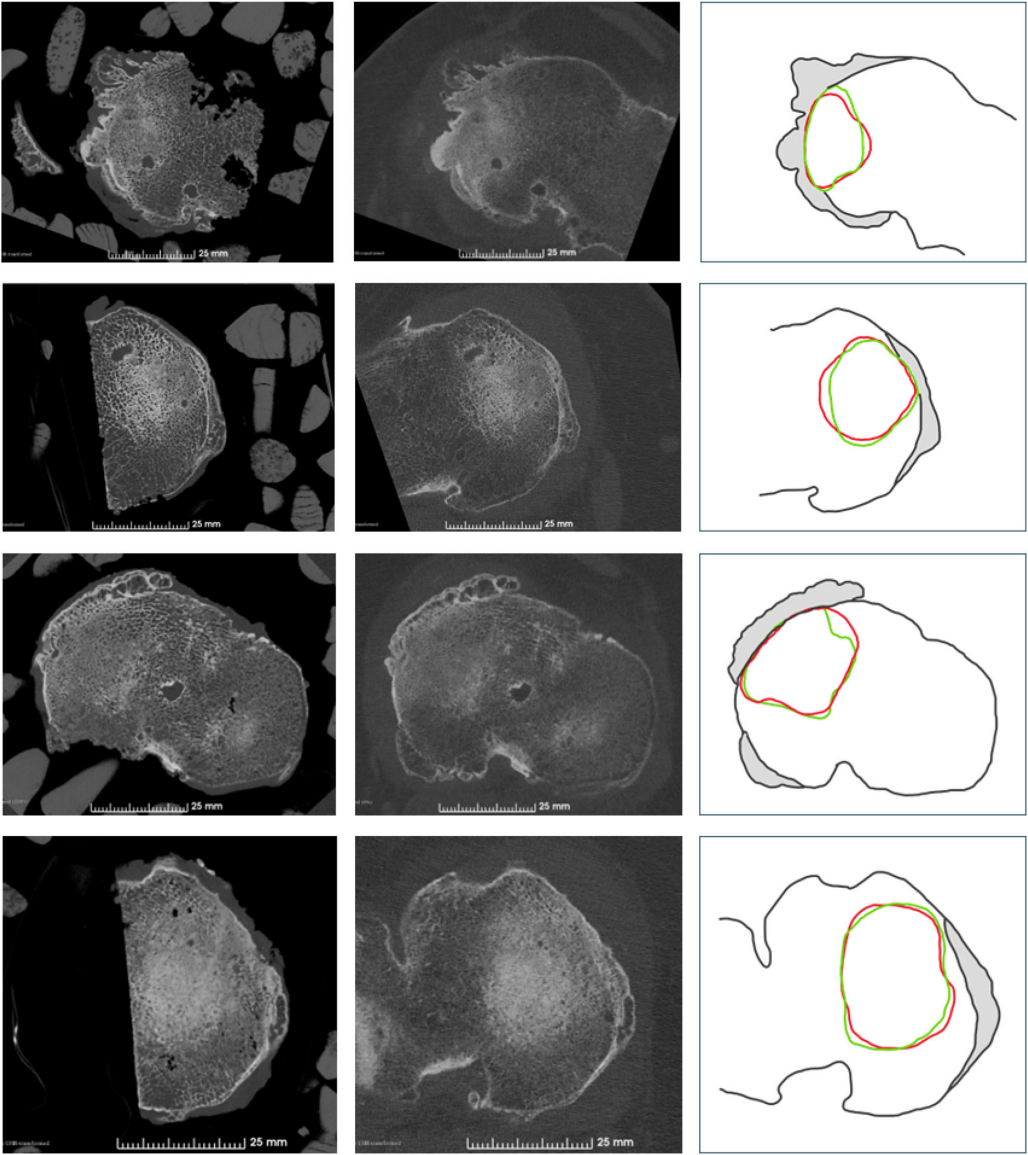
Four cases from the study. Left column: PCCT axial slice of the ex vivo tibial plateau specimen in dry ice. Middle column: PCCT in vivo axial slice of tibial plateau corresponding to ex vivo placement. Right column: Schematic drawing of the ex vivo segmentation (green) and in vivo segmentation (red) used to calculate the Dice coefficient and Hausdorff distance

Quantitative: The mean Dice coefficient for the segmented area of subchondral sclerosis in the in vivo and ex vivo PCCT was 0.91, and the mean average Hausdorff distance between in vivo and ex vivo segmentations was 0.11 mm, with a mean 95% Hausdorff distance of 0.75 mm. Four examples showing the segmentations used, the in vivo (red) and ex vivo (green), to calculate the Dice coefficient and Hausdorff distance are presented in Fig. 6, and all cases can be found in Fig. S1 in the Supplementary material.

## Discussion

In this exploratory study of the subchondral bone of the knee, we compared in vivo and ex vivo PCCT to investigate to what degree information is lost when moving from ex vivo to in vivo imaging. We found a strong correlation for the in vivo and ex vivo BV/TV (*R*^2^ = 0.82) and attenuation measures of both healthy (*R*^2^ = 0.89) and sclerotic (*R*^2^ = 0.79) bone, while a moderate correlation was found for Tb.Th. (*R*^2^ = 0.55). BV/TV has, in the literature, shown the highest correlation when testing PCCT against higher resolution modalities such as µCT and HR-pQCT [9–11]. In contrast, the microstructure parameters, such as trabecular thickness, separation and number, are harder to obtain by PCCT, presumably due to resolution limitations in PCCT.

In the Bland–Altman plot, we observed a bias of −4.1%, indicating that BV/TV values in vivo are, on average, lower than ex vivo, Fig. 3. There was a trend towards an increase in the spread of the differences at lower BV/TV values, suggesting heteroscedasticity. With a small negative trend, meaning that as the average BV/TV increases, the in vivo measurements tended to be closer to the ex vivo values. This indicates that the difference between ex vivo and in vivo measures is more pronounced in low BV/TV regions. In vivo measured Tb.Th. were consistently lower than Tb.Th. values measured ex vivo, with a bias of −0.598 mm, Fig. 3. Again, the differences increased with higher average Tb.Th., meaning that as Tb.Th. increases, the difference between in vivo and ex vivo measurements became more pronounced. A proportional bias was also observed in two other studies comparing measures of Tb.Th.by PCCT and µCT, although with an overestimation of the measures [10, 12]. The authors state that PCCT may overestimate Tb.Th., compared to higher resolution modalities, due to partial volume effects, which result in a blurring of the edges of the trabeculae, leading to a false increase in the measured thickness [12, 13]. However, in our study, in vivo measures were, on average, smaller than the ex vivo measures. Whether a lower spatial resolution and increased noise in the in vivo scan could lead to the misclassification of bone-containing pixels as non-bone, resulting in an underestimation of BV/TV and Tb.Th. is speculative and would be dependent on the thresholding method used. The thresholding method has a significant impact on the resulting morphological bone measures since whether a pixel is categorised as bone or marrow space has a direct impact on both BV/TV and Tb.Th. Although the Otsu method for thresholding is common practice for segmenting bone, perhaps using a more complex segmentation method, e.g., training a neural network, could reduce the impact of potential segmentation errors on the morphological measures. Studying different reconstruction parameters prior to thresholding could prove valuable. Future studies could investigate whether reconstruction with sharper kernels and virtual monoenergetic imaging yields a better definition of the bone trabeculae.

The small bias observed for the attenuation values could be due to beam hardening effects, which will be more pronounced in the in vivo scan and can cause lower apparent density [14], Fig. 4. The CT protocols were designed to be used in vivo, with an emphasis on reducing the effect of cupping-artefact and beam hardening artefacts; the scan protocol was not specifically designed to study ex vivo tissue samples.

Areas of subchondral bone sclerosis segmentations showed a high Dice coefficient (0.91) and low Hausdorff distance (average Hausdorff distance = 0.11 mm and 95% Hausdorff distance = 0.75 mm). Even though we acknowledge the subjective nature of these measures, subchondral sclerosis is a key radiological feature in OA, and we found it important to evaluate how this feature translated from higher resolution ex vivo scans to the scans we can achieve in vivo, considering acceptable radiation dose, soft tissue, and movement artefacts. No good definition of subchondral sclerosis exists. Most likely, the apparent thickening of the bone is the result of several different changes to the structure of the subchondral bone, occurring both in the trabecular bone and overlying bone plate [15]. On CT, there is no clear division between non-sclerotic and sclerotic bone; rather, it seems to be a gradual transition from healthy to diseased tissue. Borrowing the nomenclature from high-resolution chest CT, the pattern observed in the PCCT can be described as moving from a normal, well-organised, high-density trabecular pattern with dark intertrabecular marrow space to a more ground glass appearance with an increased density in the marrow space perhaps due to unorganised mineralisation of the marrow space [16], to lastly, the complete disappearance of the trabecular structure akin to consolidation, Fig. 5.

To our knowledge, this is the first study to compare ex vivo PCCT with in vivo clinical PCCT to study bone structure. From ex vivo studies at the wrist [9, 12, 17] and the knee [11], it is evident that the overall correlation between PCCT and HR-pQCT and µCT for the morphological bone parameters is good; however, with substantial measurement error and large mean differences [10]. To better understand the clinical implications of changes to the morphological bone parameters and their impact on bone quality, it is essential to be able to study these parameters in longitudinal studies in both healthy and diseased populations. With that, it becomes less critical if a constant bias exists; rather, the reliability and repeatability to capture true changes in an individual over time are of importance, factors we have not explored in the present study. Longitudinal studies of bone mineral density and sclerotic area demarcation would yield similar results with in vivo and ex vivo scans based on the results presented. However, when trabecular thickness or bone volume fraction is studied, it may be appreciated that the measurements might be non-linear. Therefore, in longitudinal designs, we would suggest using either paired analysis or non-parametric testing.

Some limitations should be addressed. First, we did not have histological specimens to serve as a ground truth for the outcomes. However, with this study, we did not wish to quantify the accuracy of the measures; rather, we wanted to explore the agreement between the in vivo and ex vivo scans. Second, the participants included in this study were from a randomised trial and selected based on predefined criteria. As participants were included upon referral to knee arthroplasty surgery, all had severe OA, and no healthy specimens were included. In addition, all participants had a BMI > 30 kg/m^2^, which negatively impacted the quality of the in vivo scans compared to a population of varying body weight. However, in a population of patients with knee OA, a higher mean body weight is to be expected. Third, registration was done using semi-automated approaches, and the exact slice position cannot be completely ensured. Lastly, no good definition of subchondral sclerosis exists, making it difficult to measure and compare objectively. Therefore, we present all segmentations used to calculate the Dice coefficient and Hausdorff distance in Fig. S1 for the reader to assess.

Acknowledging the exploratory nature of this study and the need for further research on reliability and repeatability, important insights concerning bone features relevant to OA do not seem to be lost when moving from ex vivo to in vivo PCCT imaging.

In conclusion, we found that in patients with severe knee OA, bone volume fraction and attenuation can be obtained with a high correlation and a small bias between in vivo and ex vivo scans, while measures of trabecular thickness only have a moderate correlation and larger bias. It shows that longitudinal studies using in vivo PCCT are feasible, but more caution may be advised when measuring trabecular thickness. Notably, the key OA feature of subchondral bone sclerosis is well translated from ex vivo to in vivo PCCT scans.

## Supplementary information

ELECTRONIC SUPPLEMENTARY MATERIAL

## Abbreviations

BV/TV: Bone volume fraction
CT: Computed tomography
HR-pQCT: High-resolution peripheral quantitative computed tomography
HU: Hounsfield units
OA: Osteoarthritis
PCCT: Photon-counting computed tomography
ROI: Regions of interest
Tb.Th.: Trabecular thickness
UHR: Ultra-high resolution
μCT: Micro computed tomography
