# Supplementary material for: Agreement between in vivo and ex vivo photon-counting CT measurements of subchondral trabecular bone features in patients with knee osteoarthritis
Source: Eur Radiol. 2025 Aug 27;36(3):2125–34. doi: 10.1007/s00330-025-11948-9 (PMC12963185; doi:10.1007/s00330-025-11948-9)
Supplement: Supplementary file 1 — ELECTRONIC SUPPLEMENTARY MATERIAL [file 330_2025_11948_MOESM1_ESM.pdf]

Agreement Between in vivo and ex vivo Photon-Counting CT Measurements of Subchondral Trabecular Bone Features in Patients with Knee Osteoarthritis

ELECTRONIC SUPPLEMENTARY MATERIAL

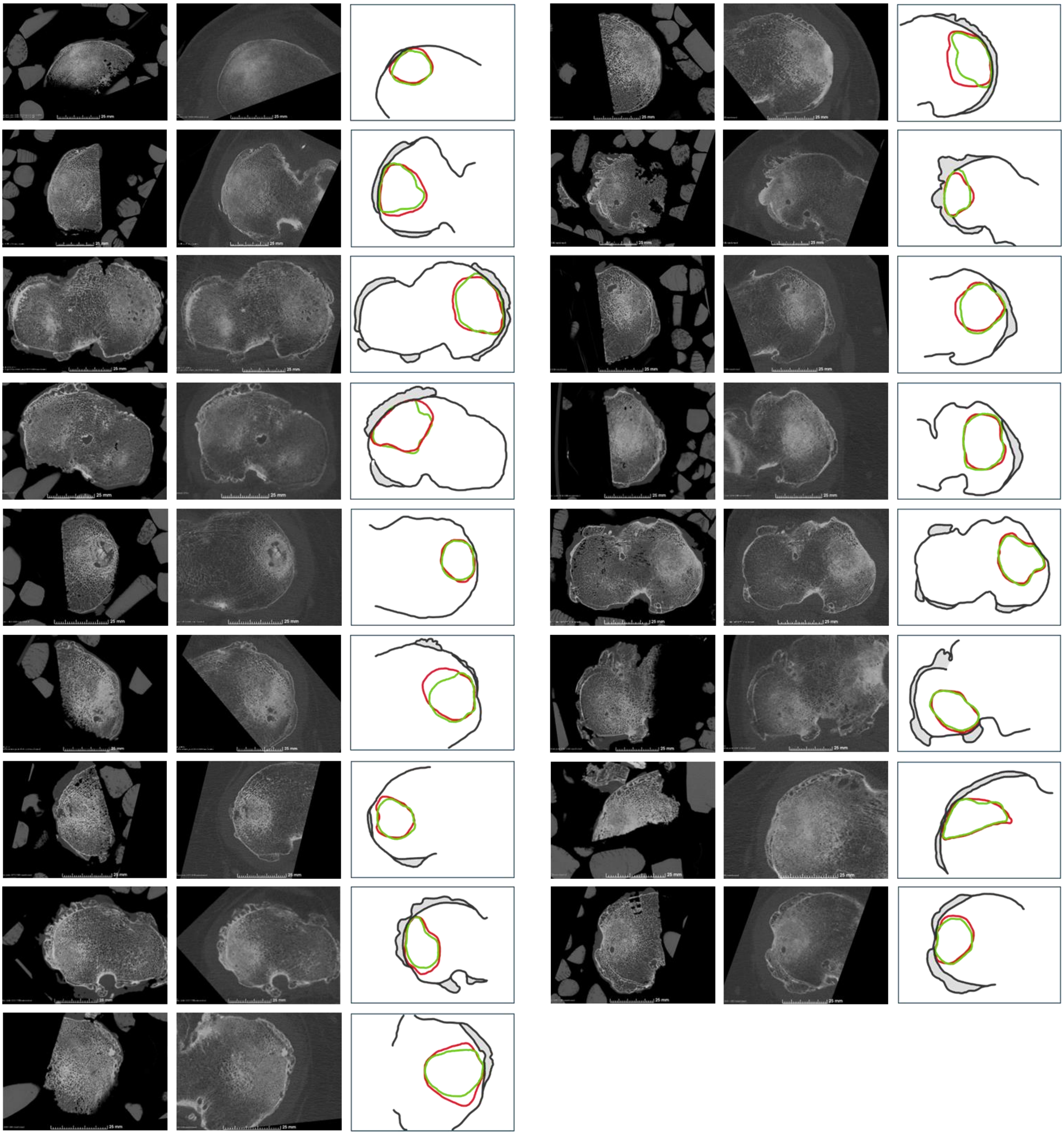

Figure S1: All cases from the study (except one, data was lost before creating the figure). Left column: Photon Counting CT axial slice of the ex vivo tibial plateau specimen in dry ice. Middle column: Photon Counting CT in vivo axial slice of tibial plateau corresponding to ex vivo placement. Right column: Schematic drawing of the ex vivo segmentation (green) and in vivo segmentation (red) used to calculate dice coefficient and Hausdorff distance.
